# Virus replicon particles expressing porcine reproductive and respiratory syndrome virus proteins elicit immune priming but do not confer protection from viremia in pigs

**DOI:** 10.1186/s13567-016-0318-0

**Published:** 2016-02-19

**Authors:** Melanie Eck, Margarita García Durán, Meret E. Ricklin, Samira Locher, Javier Sarraseca, María José Rodríguez, Kenneth C. McCullough, Artur Summerfield, Gert Zimmer, Nicolas Ruggli

**Affiliations:** Institute of Virology and Immunology IVI, Sensemattstrasse 293, 3147 Mittelhäusern, Switzerland; Graduate School for Cellular and Biomedical Sciences, University of Bern, 3012 Bern, Switzerland; Inmunología y Genética aplicada, S.A. (INGENASA), Calle de Los Hermanos García Noblejas 39, 28037 Madrid, Spain; Department of Infectious Disease and Pathobiology, Vetsuisse Faculty, University of Bern, Länggassstrasse 122, 3001 Bern, Switzerland

## Abstract

Porcine reproductive and respiratory syndrome virus (PRRSV) is the causative agent of one of the most devastating and economically significant viral disease of pigs worldwide. The vaccines currently available on the market elicit only limited protection. Recombinant vesicular stomatitis virus (VSV) replicon particles (VRP) have been used successfully to induce protection against influenza A virus (IAV) in chickens and bluetongue virus in sheep. In this study, VSV VRP expressing the PRRSV envelope proteins GP5, M, GP4, GP3, GP2 and the nucleocapsid protein N, individually or in combination, were generated and evaluated as a potential vector vaccine against PRRSV infection. High level expression of the recombinant PRRSV proteins was demonstrated in cell culture. However, none of the PRRSV antigens expressed from VRP, with the exception of the N protein, did induce any detectable antibody response in pigs before challenge infection with PRRSV. After challenge however, the antibody responses against GP5, GP4 and GP3 appeared in average 2 weeks earlier than in pigs vaccinated with the empty control VRP. No reduction of viremia was observed in the vaccinated group compared with the control group. When pigs were co-vaccinated with VRP expressing IAV antigens and VRP expressing PRRSV glycoproteins, only antibody responses to the IAV antigens were detectable. These data show that the VSV replicon vector can induce immune responses to heterologous proteins in pigs, but that the PRRSV envelope proteins expressed from VSV VRP are poorly immunogenic. Nevertheless, they prime the immune system for significantly earlier B-cell responses following PRRSV challenge infection.

## Introduction

Infections with porcine reproductive and respiratory syndrome virus (PRRSV) causes reproductive failures in sows [1] and respiratory disorders particularly in young pigs [2], which results in important economic losses worldwide [3, 4]. Recently, highly pathogenic PRRSV strains have emerged in China [5] and Eastern Europe [6]. PRRSV is an enveloped positive sense single-stranded RNA virus belonging to the family *Arteriviridae* within the order *Nidovirales* [7]. Two PRRSV genotypes can be distinguished, type 1 PRRSV of European origin and type 2 PRRSV originating from North America and China, both spreading worldwide with high genetic and antigenic diversity [8, 9]. The PRRSV genome consists of at least 10 open reading frames (ORF). ORF 1a and 1b encode the non-structural proteins from two polyproteins pp1a and pp1ab that are further processed proteolytically, as well as two proteins nsp2TF and nsp2N resulting from ribosomal frameshifts within the nsp2 gene (for a detailed review see [10]). The remaining ORFs encode the structural proteins on subgenomic messenger RNAs. ORF 2a, 2b and 3–7, encode the glycoprotein 2 (GP2) also termed GP2a, the non-glycosylated protein 2b also termed E, the glycoproteins GP3, GP4, GP5, the non-glycosylated membrane protein M (from ORF6) and the nucleocapsid protein N (from ORF7), respectively (reviewed in [11]). Recently, an alternative ORF5a protein was identified as a minor component of the equine arteritis virus (EAV) [12] and the PRRSV virions [13]. GP5 and M form a disulphide-linked heterodimer that is essential for the formation of infectious particles [14, 15]. For EAV, the glycoproteins GP2, GP3 and GP4 form a heterotrimeric complex that is stabilised by disulphide bonds, which has not been demonstrated for PRRSV yet (reviewed in [11]). The PRRSV GP5–M and GP2–GP3–GP4 complexes are linked essentially through non-covalent interactions between GP5 and GP4 [16]. The basic protein N associates with the viral RNA genome to form the nucleocapsid. N is the most immunogenic PRRSV structural protein. It elicits a strong antibody response a few days post infection (pi). These antibodies do however not neutralize the virus and are therefore not protective [17]. The major neutralizing epitopes are found on GP5 [18–21] and GP4 [22–24] which are also the most diverse structural proteins between isolates [25]. Neutralizing epitopes were also found on M, GP3 and GP2 [26–28], but their contribution to protection is unclear. GP5 co-expressed with M elicits a better neutralizing Ab response than GP5 alone [29, 30]. However, neutralizing antibodies appear typically several weeks only after the onset of the first antibody response, simultaneously with clearance of the virus from the bloodstream [21, 31].

The development of vaccines against PRRSV has been only partially successful so far and remains a challenging task (for comprehensive reviews, see [32–34]). There are currently two types of PRRSV vaccines on the market: modified live-virus vaccines (MLV) and inactivated vaccines [35–37]. MLV are typically more efficacious than inactivated vaccines [38, 39]. Numerous alternative PRRSV vaccine approaches have been explored with limited success so far (reviewed in [32, 40]). These efforts include for instance DNA vaccines, subunit and peptide vaccines, viral vector vaccines and plant-derived vaccines [30, 41–46].

Propagation-incompetent recombinant vesicular stomatitis virus (VSV) represents yet another vector vaccine approach. Recombinant VSV replicons lacking the VSV glycoprotein (G) gene and carrying genes of interest instead can be packaged in virus replicon particles (VRP) with high infectious titres using a complementing G-expressing cell line [47]. Such VRP were used successfully in the past to induce protection against SARS coronavirus in a mouse model [48], influenza A virus (IAV) in chickens and mice [49–51], and bluetongue virus (BTV) in sheep [52]. VSV VRP are safe due to the lack of glycoprotein G expression, preventing assembly and spread of virus particles. Pigs have typically no pre-existing immunity against VSV. Thus, VSV replicons represent an attractive novel vaccine platform for pigs. They have however not been evaluated in pigs yet. In the present study, VSV VRP were generated to express different combinations of the major and minor PRRSV glycoproteins. The immunogenicity and protective potential of these VRP were assessed in pigs.

## Materials and methods

### Cells and virus

MARC-145 cells (ATCC, LGC Standards) were grown in Dulbecco’s modified Eagle medium (DMEM; Life Technologies) supplemented with 10% foetal bovine serum (FBS; Biowest). BHK-21 cells were obtained from the German Cell Culture Collection (DSZM) and grown in Earle’s minimal essential medium Eagle (MEM; Life Technologies) supplemented with 5% FBS. BHK-G43, a transgenic BHK-21 cell clone expressing the VSV G protein in a regulated manner, were maintained as described previously [47]. The type 1 PRRSV strain Olot/91 was kindly provided by Luis Enjuanes (Centro Nacional de Biotecnología, Madrid, Spain). This virus was a MARC-145-adapted variant of the original Olot/91 virus and was therefore propagated and titrated in MARC-145 cells.

### Antibodies

For the detection of PRRSV proteins, monoclonal antibody (mAb) 13E2 directed against PRRSV N, and mAb VII2D directed against amino acids 73–84 of PRRSV GP3 were kindly provided by Hans Nauwynck (University Ghent, Belgium). A polyclonal rabbit serum against PRRSV GP4 was obtained from Luis Enjuanes (Centro Nacional de Biotecnología, Madrid, Spain). This serum was generated at BioGenes (Germany) with purified recombinant GP4 from PRRSV Olot/91 expressed with the baculovirus system. The mAb 11E10C7 directed against PRRSV M, and the mAb 3AH9 against PRRSV GP5 were a gift from INGENASA (Madrid, Spain). The rabbit anti-Myc antiserum C3956, the mouse anti-Flag M2 antibody and the anti-α-tubulin mAb were purchased from Sigma-Aldrich. The mouse anti-HA antibody 12CA5 was from Roche. The secondary antibodies goat anti-mouse IgG Alexa 546 and goat anti-rabbit IgG Alexa 546 were from Molecular Probes. The anti-swine IgG antibody conjugated with rhodamine was purchased from Rockland. The polyclonal rabbit anti-mouse IgG coupled with horseradish peroxidase was from DAKO.

### Construction of recombinant VSV replicon particles

The cDNA of GP5 and M and the codon-optimized cDNA of GP2, GP3 and GP4 from the PRRSV Olot/91 strain (GenBank Accession Number KC862570) were derived from pSL-ORF5–ORF6 and pSL-GP3–2–4, respectively, kindly provided by Luis Enjuanes (Centro Nacional de Biotecnología, Madrid, Spain). The codon-optimized cDNA of N from the Olot/91 strain (GenBank Accession Number AGW23409) was synthesized by GenScript (Piscataway). For generation of recombinant VSV replicons, GP5 or N were inserted into the plasmid pVSV* using *MluI* and *BstEII* restriction sites upstream and downstream of the fourth transcription unit, replacing the G gene of VSV in analogy to a previous report [49]. This replicon contained an additional transcription unit at position 5, expressing the green fluorescent protein (GFP) referred to by an asterisk (*) in the vector nomenclature. The resulting plasmids were designated pVSV*ΔG(GP5) and pVSV*ΔG(N), respectively. For generation of a dual antigen expression vector, the M cDNA was inserted into pVSV*ΔG(GP5) using *XhoI* and *NheI* restriction sites, thereby replacing the GFP gene in the fifth transcription unit. The resulting plasmid was designated pVSVΔG(GP5/M). For generation of a triple antigen expression vector, the cDNA of GP4, GP3 and GP2 was inserted into a VSVΔG vector containing 7 transcription units described recently [53] using the *MluI* (in case of GP4), *XhoI* (in case of GP3) and *NheI* (in case of GP2) restriction sites. The resulting plasmid was designated pVSVΔG(GP4/GP3/GP2). For expression of a modified M and GP3 containing a short peptide epitope at the C terminus, the M and GP3 gene, respectively, were inserted without stop codon into the pCMV-3Tag-3A plasmid vector (Agilent Technologies) upstream and in frame with a triple Flag epitope (DYKDDDDK)-coding region followed by a stop codon. The M-Flag and GP3-Flag open reading frames were amplified by PCR and inserted into the fourth transcription unit of pVSV*ΔG, resulting in the plasmids pVSV*ΔG(M-Flag) and pVSV*ΔG(GP3-Flag), respectively. The antigens GP5 and GP4 were modified by fusing a short HA epitope (YPYDVPDYA) to the C terminus, while GP2 was modified at the C terminus with a short Myc epitope (EQKLISEEDL). The ORFs were inserted into the fourth transcription unit of pVSV*ΔG resulting in the plasmids pVSV*ΔG(GP5-HA), pVSV*ΔG(GP4-HA), and pVSV*ΔG(GP2-Myc), respectively. For expression of the GP3 ectodomain (GP3ecto) without the C-terminal transmembrane domain, a gene cassette encoding the amino acids 29–176 of GP3 (numbering according to GenBank Accession Number AGW23404) was inserted in frame with the Igκ leader sequence and an optimal signalase cleavage site in the mammalian expression vector pSecTag-2A (Invitrogen). In this way GP3ecto was fused to a Myc peptide epitope and a histidine tag (6xHis) at the C terminus, followed by a stop codon. This Igκ-GP3ecto-Myc-6xHis construct was then amplified by PCR and inserted into the fourth transcription unit of pVSV*ΔG, resulting in the plasmid pVSV*ΔG(GP3ecto-Myc). For expression of IAV proteins, the cDNA encoding HA, NA, NP and M2 of IAV A/swine/Belzig/2/01 (H1N1) were kindly provided by Jürgen and Olga Stech (Friedrich-Loeffler-Institut, Greifswald-Insel Riems, Germany). The genes were inserted into the plasmid pVSV* using the *MluI* and *BstEII* restriction sites, replacing the G gene of VSV as described [49]. All nucleotide sequences were confirmed by Sanger sequencing. The recombinant replicons were propagated in the BHK-G43 helper cell line providing the VSV G protein in trans, yielding infectious VRP with titres of 10^7^–10^8^ infectious units (IU)/mL as described previously [54]. For the titrations, GFP expression was used as readout. For the detection of VRP that did not express GFP, infected cells were fixed with PBS containing 3% paraformaldehyde (PFA) for 20 min, washed with PBS containing 0.1 M (w/v) glycine and then permeabilized with 0.25% (v/v) Triton X-100. The cells were incubated with a rabbit anti-VSV serum and subsequently with a goat anti-rabbit horseradish peroxidase conjugate (DAKO) and stained with 3-amino-9-ethylcarbazole (AEC)/H_2_O_2_ as substrate. An overview of all constructed VSV VRP is provided in Table 1.

**Table 1:** VSV VRP expressing PRRSV structural proteins.

PRRSV Olot/91 antigen	VSV VRP
GP5	VSV*ΔG(GP5)^a^
GP5-HA	VSV*ΔG(GP5-HA)
M-Flag	VSV*ΔG(M-Flag)
GP5, M	VSVΔG(GP5/M)
GP2-Myc	VSV*ΔG(GP2-Myc)
GP3-Flag	VSV*ΔG(GP3-Flag)
GP3ecto	VSV*ΔG(GP3ecto-Myc)
GP4-HA	VSV*ΔG(GP4-HA)
GP4, GP3, GP2	VSVΔG(GP4/GP3/GP2)
N	VSV*ΔG(N)

^a^The * indicates the presence of the GFP gene in the vector.

### Immunofluorescence

MARC-145 cells grown on 12-mm-diameter cover slips were inoculated for 90 min at 37 °C with recombinant VRP using a multiplicity of infection (MOI) of 3 IU/cell. At 6 h post infection, the cells were fixed with 3% PFA for 20 min and washed with PBS containing 0.1 M (w/v) glycine. The cells were permeabilized with 0.25% (v/v) Triton X-100 for 5–10 min and subsequently incubated with primary and secondary antibodies, diluted in PBS containing 1% bovine serum albumin (BSA). After each incubation period (60 min, room temperature), the cells were washed three times with PBS. Finally, the cells were washed with distilled water and embedded in Mowiol 4-88 mounting medium (Sigma-Aldrich).

### Western blot

Confluent MARC-145 cells in 6-well tissue culture plates were inoculated for 90 min at 37 °C with the VRP and further incubated for 6–8 h. The cells were then washed with cold PBS and lysed with Nonidet-P40 buffer (50 mM Tris, pH 7.5, 150 mM NaCl, 1% NP-40) supplemented with protease inhibitor cocktail (Sigma-Aldrich). Proteins were dissolved in sodium dodecyl sulphate (SDS) sample buffer (125 mM Tris-HCl, pH 6.8, 20% glycerol, 1% bromophenol blue, 4% SDS) with or without 5% (v/v) β-mercaptoethanol (βME), separated (2 µg protein/lane) by SDS 12% polyacrylamide gel electrophoresis (PAGE), and transferred to nitrocellulose membranes by semi-dry blotting according to standard protocols. The nitrocellulose membranes were blocked overnight at 4 °C with Odyssey Blocking Reagent (LI-COR Biosciences) diluted 1:2 with PBS. For immunodetection, the membranes were incubated with primary and IRDye-conjugated secondary antibodies, diluted in Odyssey Blocking Reagent/PBS (1:2). After each incubation period (60 min, room temperature), the membranes were washed with PBS supplemented with 0.1% Tween-20. Finally, the membranes were washed twice in detergent-free PBS and images acquired with the Odyssey Infrared Imaging System (LI-COR Biosciences).

### Experimental design of animal studies

All pigs were obtained from the specific pathogen free (SPF) breeding facility of the Institute of Virology and Immunology IVI. Three experiments were performed. For each experiment the pigs were randomly assigned to treatment groups housed in separate stables. The groups were immunized by intramuscular injection of 5 mL of cell culture supernatant containing 10^7^–10^8^ IU/mL recombinant VRP. In the first experiment, 2 groups of pigs (*n* = 5) were immunized three times at 9½, 13½ and 18½ weeks of age with VSVΔG(GP5/M) or with the control VSV*ΔG VRP, respectively. In the second experiment, one group of pigs (*n* = 4) was immunized twice at 10½ and 14½ weeks of age with a mixture of VSVΔG(GP5/M) and VSVΔG(GP4/GP3/GP2) and the second group (*n* = 3) received the control VSV*ΔG VRP. In the third experiment, 4 groups of pigs (*n* = 2) were immunized three times at 5, 6 and 7 months of age with two different VSV VRP each injected at two different sites, i.e. VSV*ΔG(N) and VSV*ΔG(HA_Belz_), VSV*ΔG(GP3ecto-Myc) and VSV*ΔG(M2_Belz_), VSV*ΔG(GP4-HA) and VSV*ΔG(NA_Belz_), VSV*ΔG(control) and VSV*ΔG(NP_Belz_), respectively. In all experiments, the pigs were challenged via the intranasal route with 10^6^ TCID_50_/animal of the PRRSV strain Olot/91 3–4 weeks after the last vaccination. The challenge virus was diluted in 5 mL MEM and administered dropwise intranasally. Blood was taken before each vaccination and at regular intervals after vaccination and after the challenge infection. Serum was stored at −70 °C. Body temperature and clinical score were monitored daily according to a defined scoring system [55].

### Ethics statement

The experiments in pigs were performed in compliance with the Swiss animal protection law and approved by the animal welfare committee of the canton of Berne, Switzerland (Authorization Number BE89/11).

### PRRSV titration

Titration by end point dilution was performed in MARC-145 cells grown in 96-well plates. The cells were inoculated with tenfold serially diluted serum samples. At 48 h pi, the cells were fixed and immunoperoxidase staining with the anti-N mAb 13E2 was performed according to standard protocols. The 50% end point titre was expressed as tissue culture infectious dose 50% (TCID_50_)/mL, with a limit of detection of 1.7 log_10_ TCID_50_/mL.

### RNA extraction and quantitative RT-PCR (RT-qPCR)

Total cellular RNA was extracted from pig sera using the NucleoSpin Multi 96 Virus kit (Macherey–Nagel) on a Freedom EVO Robot (Tecan) and stored at −70 °C. For detection of viral RNA, a reverse transcriptase quantitative PCR (RT-qPCR) based on the amplification of the conserved region of ORF7 was performed in duplicates employing GFP messenger RNA as internal control [56]. The results were expressed as the total number of cycles minus the quantification cycle (Cq)-value.

### Enzyme-linked immunosorbent assay (ELISA)

The ELISA PRRS X3 (IDEXX Laboratories) was used for measuring anti-PRRSV IgG antibodies. Samples to positive (S/P) ratios higher than 0.4 were considered positive. Antibodies against PRRSV GP5, GP4 and GP3 were detected using in house competitive (GP5) and indirect (GP4 and GP3) ELISAs (INGENASA, Madrid, Spain). These ELISAs were performed with plates coated with recombinant GP5, GP4 and GP3 antigens from PRRSV Olot/91. For the GP5 ELISA, the percentage of competition was calculated using the formula [(ODneg − ODsample)/(ODneg − ODpos)] × 100. Positive and negative controls were provided by INGENASA (Spain). Samples were considered positive if the percentage of competition was >37%. For the indirect ELISAs, appropriate cut-offs were set. For the detection of C-reactive protein (CRP; Genway), haptoglobin (Hp; Genway) and IFN-γ (Mabtech) in pig sera, commercially available ELISAs were used according to the manufacturer’s protocols. Porcine interferon-α (IFN-α) was determined by ELISA as described previously [57].

### Virus neutralization assay

Serum samples were heat inactivated at 56 °C for 30 min prior to performing the serum neutralization assay. 50 µL of two-fold serially diluted sera were mixed with an equal volume of 100 TCID_50_ of PRRSV Olot/91 and incubated in 96-well tissue culture plates for 1 h at 37 °C. After 1 h, 10^4^ MARC-145 cells were added to each well. After 6 days, the culture plate was fixed with 3% PFA. The presence of virus was detected by CPE and by staining with the anti-N mAb and AEC/H_2_O_2_. The virus neutralization titre was expressed as the reciprocal of the serum dilution leading to 50% reduction of infection.

### Statistical analysis

Data were analysed with the GraphPad Prism 6.01 software. Significant differences between groups were assessed by multiple *t* tests. *P* < 0.05 was considered significant.

## Results

### Expression of PRRSV structural proteins using VSV VRP

In previous work, a propagation-incompetent VSV replicon vector was generated by replacing the gene of the glycoprotein G in the fourth transcription unit of the viral genome with influenza virus genes. This replicon contained the GFP gene [referred to by an asterisk (*) in the vector nomenclature] in an additional transcription unit at position 5 [49]. Based on this vector, we generated several VSV VRP expressing the individual structural antigens of PRRSV Olot/91 or combinations thereof for evaluation as PRRS vaccine candidates (see “Materials and methods”; Table 1). The empty parental VSV*ΔG vector served as control [49].

**Figure 1:**
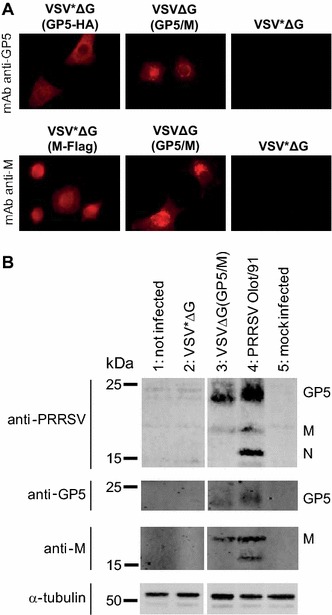
**Characterization of VRP-mediated expression of GP5 and M.** **A** Immunofluorescence analysis of MARC-145 cells 6 h after infection with VSV*ΔG(GP5-HA), VSV*ΔG(M-Flag), VSVΔG(GP5/M) or with the VSV*ΔG control VRP. In the top panels, expression of GP5 is detected using the anti-GP5 mAb 3AH9. In the bottom panels, expression of M is detected using the anti-M mAb 11E10C7. **B** Western blot analysis of lysates from non-infected cells (lane 1) or cells infected with VSV*ΔG (lane 2), VSVΔG(GP5/M) (lane 3), PRRSV Olot/91 (lane 4) or mock (lane 5). The proteins were separated by SDS-PAGE under reducing (+βME) conditions and blotted onto a nitrocellulose membrane. The GP5 and M proteins were detected with the anti-GP5 and anti-M mAbs and with a porcine anti-PRRSV serum that reacted also with N. α-tubulin served as loading control using the anti-α-tubulin mAb. Protein molecular weight (kDa) standards are indicated.

The VSV VRP-mediated expression of the recombinant PRRSV antigens was studied in MARC-145 cells, taking advantage of the broad tropism of the VSV particles. This allowed direct comparison of the recombinant proteins with the viral proteins in PRRSV-infected MARC-145 cells. GP5 was detected by immunofluorescence with the anti-GP5 mAb in cells infected with either VSV(GP5-HA) or VSV(GP5/M), but not in cells infected with the parental VSV*ΔG (Figure 1A). The anti-M mAb reacted specifically with cells infected with VSV*ΔG(M-Flag) or with VSVΔG(GP5/M). In Western blots, porcine anti-PRRSV serum reacted with 24 and 18 kDa proteins from VSVΔ(GP5/M)-infected cells, representing GP5 and M, respectively (Figure 1B). PRRSV Olot/91-infected cells served as positive control showing expression of GP5, M and N. Under non-reducing conditions, porcine anti-PRRSV serum revealed a protein of higher molecular weight, indicating disulphide-linked association of GP5 and M (not shown). Expression of GP5 and M of the expected molecular weight was confirmed in Western blots with the anti-GP5 and anti-M mAbs, respectively (Figure 1B). VSV*ΔG-infected cells were used as a negative control and did not show any expression of PRRSV proteins. These results show that the PRRSV GP5 and M antigens could be successfully co-expressed from a single VSV replicon vector.

**Figure 2:**
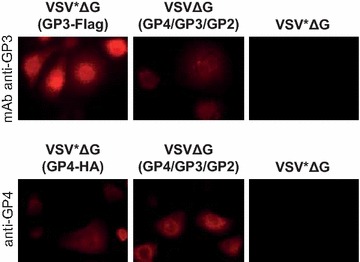
**Characterization of VRP-mediated expression of GP3 and GP4.** Immunofluorescence analysis of MARC-145 cells 6 h after infection with VSV*ΔG(GP3-Flag), VSV*ΔG(GP4-HA), VSVΔG(GP4/GP3/GP2) or with the VSV*ΔG control VRP. Expression of GP3 (top panels) and GP4 (bottom panels) were detected with the anti-GP3 mAb VII2D and with a rabbit anti-GP4 serum, respectively.

MARC-145 cells infected with either VSV*ΔG(GP3-Flag) or VSVΔG(GP4/GP3/GP2) reacted specifically with the anti-GP3 mAb, while cells infected with either VSV*ΔG(GP4-HA) or VSVΔG(GP4/GP3/GP2) reacted with a polyclonal anti-GP4 immune serum (Figure 2). Since a specific antibody against GP2 was not available, the expression of GP2 could not be demonstrated. Any tag was omitted on purpose in the VSVΔG(GP4/GP3/GP2) to preserve the natural conformation of the three proteins. Nevertheless, Myc-tagged GP2 expression was detected from VSV*Δ(GP2-Myc) using the anti-Myc serum (not shown).

**Figure 3:**
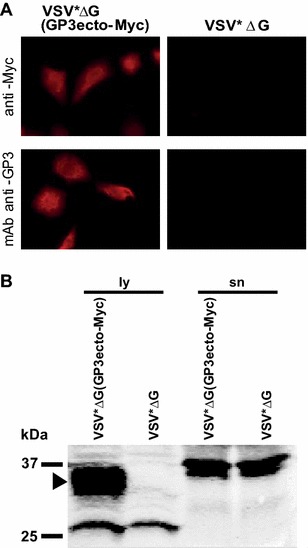
**Characterization of VRP-mediated expression of the GP3 ectodomain.** **A** Immunofluorescence analysis of MARC-145 cells 6 h after infection with VSV*ΔG(GP3ecto-Myc) or with the VSV*ΔG control. Expression of GP3ecto is detected with a rabbit anti-Myc serum (top panels) and with the anti-GP3 mAb VII2D (bottom panels). **B** Western blot analysis of lysates (ly) and supernatants (sn) from cells infected with VSV*ΔG(GP3ecto-Myc) separated by SDS-PAGE under non-reducing (-βME) conditions, using the anti-Myc serum for GP3ecto-Myc detection (arrowhead). Protein molecular weight (kDa) standards are indicated.

With the aim of evaluating whether protein secretion into the supernatant may enhance B-cell responses, the VSV*ΔG(GP3ecto-Myc) replicon was constructed for the expression of the ectodomain of GP3 fused to a Igκ leader sequence and an optimal signalase cleavage site. The Myc-tagged ectodomain of GP3 reacted with the anti-GP3 mAb and anti-Myc serum as expected (Figure 3A). Western-blot analysis with the anti-Myc serum demonstrated the presence of a 35 kDa protein in the cell lysate (ly) which was however missing in the supernatant (sn) of VSV*ΔG(GP3ecto-Myc) infected MARC-145 cells (Figure 3B). This showed that the ectodomain of GP3 was retained in intracellular compartments despite the Igκ signal sequence and the lack of the transmembrane domain.

**Figure 4:**
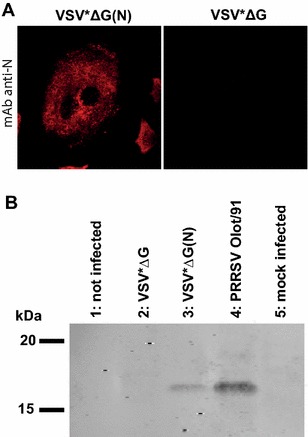
**Characterization of VRP-mediated expression of N.** **A** Immunofluorescence analysis of MARC-145 cells 6 h after infection with VSV*ΔG(N) or with the VSV*ΔG control. The expression of N is detected with the anti-N mAb 13E2. **B** Western blot analysis of lysates from non-infected cells (lane 1) or cells infected with VSV*ΔG (lane 2), VSVΔ*G(N) (lane 3), PRRSV Olot/91 (lane 4) or mock (lane 5). The proteins were separated by SDS-PAGE under non-reducing (-βME) conditions and blotted onto a nitrocellulose membrane. N antigen was detected with the anti-N mAb as above. Protein molecular weight (kDa) standards are indicated.

Finally, expression of the PRRSV N antigen by VSV*ΔG(N) was demonstrated by immunofluorescence (Figure 4A) and by Western blot (Figure 4B) using the anti-N mAb. A 16 kDa protein was detected in the lysate of both, VSV*ΔG(N)- and PRRSV Olot/91-infected cells.

### Immunization of pigs with VSV VRP co-expressing GP5 and M primes the pigs for earlier antibody responses against challenge virus infection but does not protect from viremia

The GP5 and M complex constitutes the major protein component of the PRRSV envelope against which neutralizing antibodies are formed [18, 19, 26]. Therefore, we first determined the immunogenicity of GP5/M-recombinant VSV replicons in pigs. Two groups of five pigs were immunized three times with VSVΔG(GP5/M) or with the control VSV*ΔG respectively, and were challenged 3 weeks later with the homologous PRRSV Olot/91 strain.

**Figure 5:**
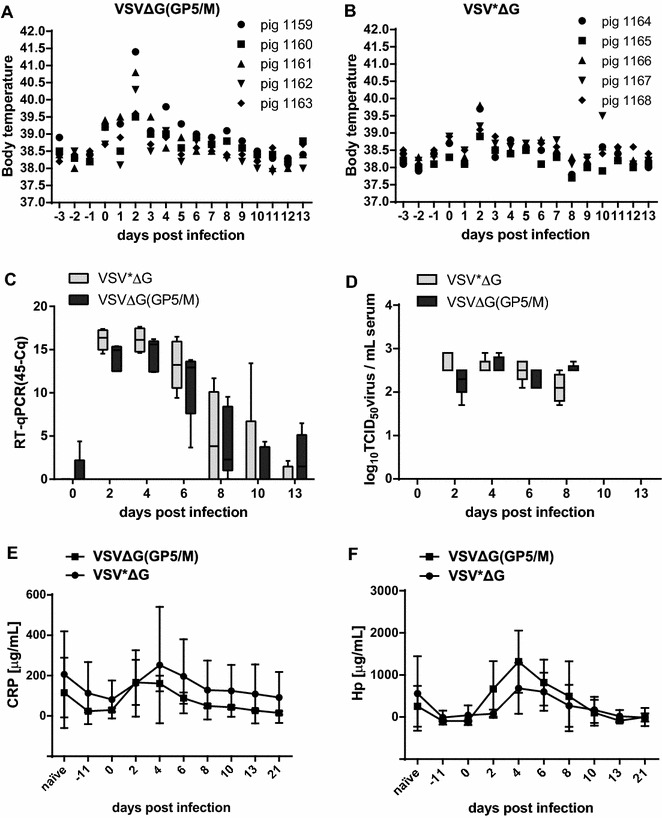
Body temperatures, virus load and acute phase proteins after challenge infection of pigs vaccinated with VSVΔG(GP5/M) or with VSV*ΔG as control. Two groups of 5 SPF pigs each were vaccinated three times with 28 and 35 days interval, with 10^7^ PFU/pig of VSVΔG(GP5/M) or of VSV*ΔG as mock control, respectively. 4 weeks after the 3rd vaccination, the pigs were infected intranasally with 10^6^ PFU/pig of the homologous Olot/91 virus. The body temperatures **A**, **B** are shown for the individual animals. Blood was collected at the indicated days after infection. PRRSV load in serum was determined by real-time RT-qPCR (**C**) and by virus titration in MARC-145 cells (**D**), and the values expressed in 45-Cq and TCID_50_/mL serum (detection limit 1.7 log_10_ TCID_50_/mL), respectively. The serum concentrations (µg/mL) of the porcine acute phase proteins CRP (**E**) and Hp (**F**) were determined by ELISA. The data of panels **C**–**F** represent the mean of five pigs per group, with error bars representing the standard deviation (SD).

Following PRRSV challenge infection, three VSVΔG(GP5/M)-vaccinated pigs out of five developed fever (40.3–41.5 °C) on day 2 pi, whereas the control pigs had no fever despite slightly elevated body temperature (Figures 5A and B). All animals developed a low viremia of short duration (Figures 5C and D). A maximum mean virus titre of 3.0 log_10_ TCID_50_/mL was reached in the serum at day 2 post challenge. The virus was detectable up to day 8 and viral RNA up to day 13 after challenge. No significant differences in viremia and viral RNA in serum were observed between the vaccinated group and the control group at any time.

Before challenge infection, vaccination with VSVΔG(GP5/M) did not induce any detectable antibody response against GP5 as measured by ELISA (not shown). The first GP5-specific antibody responses were detected by ELISA in the VSVΔG(GP5/M)-vaccinated group in 2 out of five pigs on day 6 after the challenge, and all pigs of this group were positive on day 13 pi. All pigs of the VSV*ΔG control group remained GP5 antibody negative at this time (Table 2). In the VSV*ΔG control group, GP5-specific antibodies were detected for the first time on day 21 pi in 2 out of the 5 pigs. Seroconversion against N in response to the PRRSV challenge infection was similar in the two groups, with the first N-specific seroconversion observed on day 10 pi (Table 2). Virus neutralizing antibodies were not detected in any of the animals, neither before nor after the challenge (day 0 and day 21 pi). IFN-α and IFN-γ could not be detected in the serum at any time (data not shown). Specific T-cell responses were not detected before challenge (not shown) and were therefore not further investigated. The acute phase proteins CRP and Hp which are early and sensitive markers of disease and inflammation including PRRSV infection [58] were increased in both groups between days 2 and 6 pi (Figures 5E and F). However, no significant differences could be detected between vaccinated and control animals at any time. Thus, apart from priming pigs for GP5-specific antibody responses after challenge infection, the VSVΔG(GP5/M) vector could not induce any seroconversion against GP5 before challenge nor any sign of protection from viremia after infection.

**Table 2:** Seroconversion after vaccination of pigs with VSVΔG(GP5/M) followed by PRRSV challenge.

Ab against PRRSV Ag (by ELISA)	Vaccine	ELISA	Days after PRRSV challenge infection							
			0	2	4	6	8	10	13	21
GP5	VSVΔG(GP5/M)	Positive/total pigs	0/5	0/5	0/5	2/5	2/5	4/5	5/5	5/5
		% Competition^a^ (positive pigs)	0	0	0	50 ± 12	49 ± 29	82 ± 15	82 ± 20	69 ± 15
		Range for positive pigs	0	0	0	41–59	31–66	64–101	59–106	49–91
	VSV*ΔG (control)	Positive/total pigs	0/5	0/5	0/5	0/5	0/5	0/5	0/5	2/5
		% Competition (positive pigs)	0	0	0	0	0	0	0	62 ± 13
		Range for positive pigs	0	0	0	0	0	0	0	53–72
N	VSVΔG(GP5/M)	Positive/total pigs	0/5	0/5	0/5	0/5	0/5	2/5	4/5	4/5
		S/P ratio^b^ (positive pigs)	0	0	0	0	0	0.9 ± 0.4	1.0 ± 0.5	1.2 ± 0.1
		Range (positive pigs)	0	0	0	0	0	0.5–1.2	0.6–1.7	1.1–1.3
	VSV*ΔG (control)	Positive/total pigs	0/5	0/5	0/5	0/5	0/5	0(1)/5	4/5	3/5
		S/P ratio (positive pigs)	0	0	0	0	0	0	0.7 ± 0.2	1.4 ± 0.8
		Range (positive pigs)	0	0	0	0	0	0	0.4–0.9	0.4–1.9

IDEXX X3 kit S/P ratios of >0.4 are considered positive. The mean values are indicated with the standard deviation.

^a^% Competition = (negative control mean [mean of optical absorbance] − sample mean)/(negative control mean − positive control mean) × 100. The mean values are indicated with the standard deviation.

^b^S/P ratio = (sample mean [mean of optical absorbance] − negative control mean)/(positive control mean − negative control mean).

### Immunization of pigs by co-expression of GP5, M, GP4, GP3 and GP2 from two VSV VRP does not further enhance antibody responses nor protect against challenge virus infection

In order to determine whether the immune responses could be enhanced and induced before challenge by providing the three minor glycoproteins GP4, GP3 and GP2 together with GP5 and M, pigs (*n* = 4) were immunized twice with a mixture of VSVΔG(GP5/M) and VSVΔG(GP4/GP3/GP2) or with VSV*ΔG as control (*n* = 3) followed by PRRSV Olot/91 challenge.

**Figure 6:**
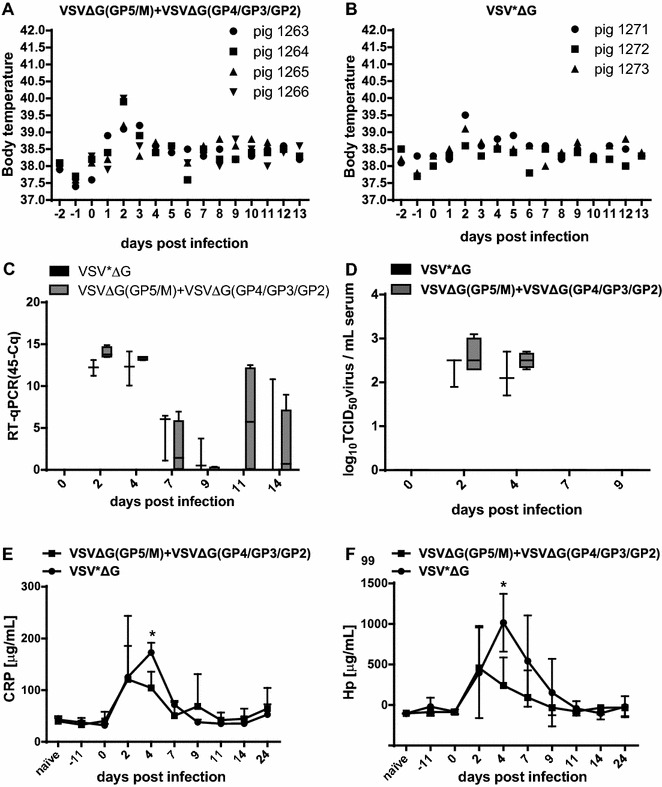
Body temperatures, virus load and acute phase proteins after challenge infection of pigs vaccinated with a mixture of VSVΔG(GP5/M) and VSVΔG(GP4/GP3/GP2) or with VSV*ΔG as control. Two groups of 4 and 3 SPF pigs were vaccinated two times at 28 days interval with a mixture of 10^7^ PFU/pig of the VSVΔG(GP5/M) and VSVΔG(GP4/GP3/GP2) VRP or with the VSV*ΔG mock control, respectively. 26 days after the 2nd vaccination, the pigs were infected intranasally with 10^6^ PFU/pig of the homologous Olot/91 virus. The body temperatures **A**, **B** are shown for the individual animals. Blood was collected at the indicated days post infection. PRRSV load in serum was determined by real-time RT-qPCR (**C**) and by virus titration in MARC-145 cells (**D**), and the values expressed in 45-Cq and TCID_50_/mL serum (detection limit 1.7 log_10_ TCID_50_/mL), respectively. The serum concentrations (µg/mL) of the porcine acute phase proteins CRP (**E**) and Hp (**F**) were determined by ELISA. The data of panels **C**–**F** represent the mean of four and three pigs per group respectively, with error bars representing the SD and asterisks showing significant differences (*p* < 0.05).

On day 2 after challenge, two out of the four vaccinated pigs developed mild fever (40 °C; Figure 6A) while all control animals remained free of fever (<40 °C; Figure 6B). All animals developed a short viremia following challenge infection with no significant differences in viral RNA load and virus titres between the vaccinated and control groups (Figures 6C and D). As in the previous experiment, a maximum mean virus titre of 3.0 log_10_TCID_50_/mL was reached at day 2 post challenge.

Again, before challenge infection, vaccination with VRP expressing the structural proteins of PRRSV did not induce any detectable antibody response against GP5, GP4 and GP3 as measured by ELISA (not shown). Following challenge infection, GP5-specific antibody responses were detected on day 7 pi in 2 out of 4 pigs, and all pigs vaccinated with VRP expressing the PRRSV proteins seroconverted to GP5 at day 11 pi (Table 3). All pigs of the VSV*ΔG control group remained negative until day 24 pi. Seroconversion to GP3 (Table 3) was detected as early as day 7 pi in 3 of 4 pigs of the vaccinated group and in all vaccinated pigs at day 9 pi, whereas pigs of the VSV*ΔG control group remained negative until day 24 pi. A GP4-specific immune response could be detected only in 2 out of 4 vaccinated animals on day 24 pi. Here also, the seroconversion against N following challenge was similar in the two groups (Table 3). None of the animals developed any neutralizing antibodies before nor after challenge (day 0 and day 24 pi). IFN-α and IFN-γ were not found in any of the sera in this experiment either (data not shown) whereas the serum CRP and Hp levels increased significantly in both groups between days 2 and 4 after challenge and declined subsequently. On day 4 pi, the CRP and Hp levels were significantly different between the vaccinated and the control group (Figures 6E and F), suggesting that the vaccinated group developed a slightly reduced inflammatory response after co-vaccination with VSVΔG(GP5/M) and VSVΔG(GP4/GP3/GP2).

**Table 3:** Seroconversion after vaccination of pigs with VSVΔG(GP5/M) + VSVΔG(GP4/GP3/GP2) followed by PRRSV challenge.

Ab against PRRSV Ag (by ELISA)	Vaccine	ELISA	Days after PRRSV challenge infection							
			0	2	4	7	9	11	14	24
GP5	VSVΔG(GP5/M) + VSVΔG(GP4/GP3/GP2)	Positive/total pigs	0/4	0/4	0/4	2/4	2/4	4/4	4/4	4/4
		% Competition^a^ (positive pigs)	0	0	0	44 ± 0.6	48 ± 4	55 ± 10	89 ± 13	89 ± 11
		Range (positive pigs)	0	0	0	43–44	45–51	45–65	72–102	77–100
	VSV*ΔG (control)	Positive/total pigs	0/3	0/3	0/3	0/3	0/3	0/3	0/3	1/3
		% Competition (positive pigs)	0	0	0	0	0	0	0	42
		Range (positive pigs)	0	0	0	0	0	0	0	42
GP3	VSVΔG(GP5/M) + VSVΔG(GP4/GP3/GP2)	Positive/total pigs	0/4	0/4	0/4	3/4	4/4	4/4	4/4	4/4
	VSV*ΔG (control)	Positive/total pigs	0/3	0/3	0/3	0/3	0/3	0/3	0/3	2/3
GP4	VSVΔG(GP5/M) + VSVΔG(GP4/GP3/GP2)	Positive/total pigs	0/4	0/4	0/4	0/4	0/4	0/4	1/4	2/4
	VSV*ΔG (control)	Positive/total pigs	0/3	0/3	0/3	0/3	0/3	0/3	0/3	1/3
N	VSVΔG(GP5/M) + VSVΔG(GP4/GP3/GP2)	Positive/total pigs	0/4	0/4	0/4	0/4	0/4	0/4	1/4	4/4
		S/P ratio^b^ (positive pigs)	0	0	0	0	0	0	0.5	1.4 ± 0.4
		Range (positive pigs)	0	0	0	0	0	0	0.5	0.9–1.9
	VSV*ΔG (control)	Positive/total pigs	0/3	0/3	0/3	0/3	1/3	1/3	1(1)/3	3/3
		S/P ratio (positive pigs)	0	0	0	0	0.5	0.7	0.8	1.4 ± 0.7
		Range (positive pigs)	0	0	0	0	0.5	0.7	0.8	0.8–2.1

^a^% Competition = (negative control mean [mean of optical absorbance] − sample mean)/(negative control mean − positive control mean) × 100. The mean values are indicated with the standard deviation.

^b^S/P ratio = (sample mean [mean of optical absorbance] − negative control mean)/(positive control mean − negative control mean). IDEXX X3 kit S/P ratios of >0.4 are considered positive. The mean values are indicated with the standard deviation.

Together, these two vaccination trials show that VSV VRP cannot induce any detectable seroconversion against PRRSV before challenge nor any protection against virus infection and viremia. Nevertheless, these vectors can clearly prime pigs for earlier PRRSV-specific antibody responses after challenge infection.

### The PRRSV envelope proteins expressed from VRP are poorly immunogenic in pigs, as opposed to influenza A virus proteins and to the PRRSV nucleocapsid protein

The poor immunogenicity of VSV VRP expressing PRRSV envelope proteins contrasts with previous reports showing protection against IAV and BTV infections in chickens and sheep, respectively [49, 50, 52]. Thus, the lack of antibody induction after immunization of pigs with VRP expressing the five PRRSV envelope proteins raises the questions whether this vector vaccine is suitable for induction of antibody responses in pigs or whether the PRRSV proteins are poorly immunogenic per se. In order to test this, the immunogenicity of PRRSV and IAV proteins expressed from VSV VRP was assessed in the same animal by co-vaccination with two different VSV VRP constructs expressing a PRRSV protein (N, GP3ecto, GP4 or empty vector) and a IAV protein (HA, M2, NA, NP), respectively, injected at two different sites (see “Materials and methods”). After 3 vaccinations, the pigs were challenged with the homologous PRRSV Olot/91 strain.

While, antibody responses were detected against all influenza virus proteins on day 14 after vaccination (including neutralizing antibodies, not shown), no seroconversion against any of the PRRSV proteins except N could be detected before challenge (Table 4). Pigs vaccinated with VSV*∆G(N) seroconverted on day 63 after vaccination (7 days after the third vaccination). Following challenge infection with PRRSV Olot/91, the control group seroconverted on day 21 pi. There was no evidence of protection from virus infection after PRRSV challenge as indicated by the kinetics of viremia (data not shown). Nevertheless, a GP3-specific immune response was induced earlier after challenge (day 14 pi) in the VSV*ΔG(GP3ecto-Myc) vaccinated pigs compared with the VSV*ΔG-vaccinated pigs (no response on day 21 pi). A GP4-specific immune response was also induced on day 14 pi whereas the VSV*ΔG-vaccinated pigs stayed seronegative against GP4. In line with the two previous immunization trials, these data show again a priming effect. More importantly however, simultaneous vaccination of the same pigs with two different VRP show clearly that the PRRSV antigens are far less immunogenic than any of the IAV antigens tested.

**Table 4:** Seroconversion after vaccination of pigs with VSV*ΔG(N), VSV*ΔG(GP3ecto-Myc), VSV*ΔG(GP4-HA) and VSV*ΔG expressing IAV proteins.

Ab against PRRSV Ag (by ELISA)	Vaccine	ELISA	Days after VRP vaccination					Days after PRRSV challenge								
			0	14	28	63^a^	77	0	2	4	7	9	11	14	21	28
N	VSV*ΔG(N)	Positive/total pigs	0/2	n.d.	0/2	2/2	2/2	n.d.	n.d.	n.d.	2/2	n.d.	n.d.	2/2	n.d.	n.d.
		S/P ratio^b^ (positive pigs)	0	n.d.	0	0.7	0.9	n.d.	n.d.	n.d.	2.3	n.d.	n.d.	3.0	n.d.	n.d.
		Range (positive pigs)	0	n.d.	0	0.6–0.7	0.8–1.0	n.d.	n.d.	n.d.	2.0–2.6	n.d.	n.d.	2.9–3.0	n.d.	n.d.
	VSV*ΔG (control)	Positive/total pigs	0/2	n.d.	n.d.	n.d.	0/2	0/2	0/2	0/2	0/2	0/2	0/2	1/2	2/2	2/2
		S/P ratio (positive pigs)	0	n.d.	n.d.	n.d.	0	0	0	0	0	0	0	0.6	0.7	0.7
		Range (positive pigs)	0	n.d.	n.d.	n.d.	0	0	0	0	0	0	0	0.6	0.6–0.7	0.7–0.8
GP3	VSV*ΔG(GP3ecto-Myc)	Positive/total pigs	0/2	n.d.	n.d.	n.d.	0/2	n.d.	n.d.	n.d.	0/2	n.d	n.d.	2/2	2/2	n.d.
	VSV*ΔG (control)	Positive/total pigs	0/2	n.d.	n.d.	n.d.	0/2	n.d.	n.d.	n.d.	0/2	n.d.	n.d.	0/2	0/2	n.d.
N	VSV*ΔG(GP3ecto-Myc)	Positive/total pigs	0/2	n.d.	n.d.	n.d.	n.d.	0/2	0/2	0/2	0/2	0/2	0/2	1/2	1/2	2/2
		S/P ratio (positive pigs)	0	n.d.	n.d.	n.d.	n.d.	0	0	0	0	0	0	0.5	1.3	1.0
		Range (positive pigs)	0	n.d.	n.d.	n.d.	n.d.	0	0	0	0	0	0	0.5	1.3	0.5–1.6
GP4	VSV*ΔG(GP4-HA)	Positive/total pigs	0/2	n.d.	n.d.	n.d.	0/2	n.d.	n.d.	n.d.	0/2	n.d.	n.d.	2/2	2/2	n.d.
	VSV*ΔG (control)	Positive/total pigs	0/2	n.d.	n.d.	n.d.	0/2	n.d.	n.d.	n.d.	0/2	n.d.	n.d.	0/2	0/2	n.d.
N	VSV*ΔG(GP4-HA)	Positive/total pigs	0/2	n.d.	n.d.	n.d.	n.d.	0/2	0/2	0/2	0/2	0/2	0/2	1(1)/2	2/2	2/2
		S/P ratio (positive pigs)	0	n.d.	n.d.	n.d.	n.d.	0	0	0	0	0	0	0.6	0.6	0.7
		Range (positive pigs)	0	n.d.	n.d.	n.d.	n.d.	0	0	0	0	0	0	0.6	0.6–0.7	0.7
HA	VSV*ΔG(HA_Belz_)	Positive/total pigs	0/2	2/2	2/2	2/2	2/2	n.a.	n.a.	n.a.	n.a.	n.a.	n.a.	n.a.	n.a.	n.a.
NA	VSV*ΔG(NA_Belz_)	Positive/total pigs	0/2	2/2	2/2	2/2	2/2	n.a.	n.a.	n.a.	n.a.	n.a.	n.a.	n.a.	n.a.	n.a.
NP	VSV*ΔG(NP_Belz_)	Positive/total pigs	0/2	2/2	2/2	2/2	2/2	n.a.	n.a.	n.a.	n.a.	n.a.	n.a.	n.a.	n.a.	n.a.
M2	VSV*ΔG(M2_Belz_)	Positive/total pigs	0/2	2/2	2/2	2/2	2/2	n.a.	n.a.	n.a.	n.a.	n.a.	n.a.	n.a.	n.a.	n.a.

n.d.: not done; n.a.: not applicable.

^a^Antibodies against GP3 and GP4 were also analysed at day 56. The results were 0/2.

^b^S/P ratio = (sample mean [mean of optical absorbance] − negative control mean)/(positive control mean − negative control mean). IDEXX X3 kit S/P ratios of >0.4 are considered positive. The S/P ratios represent the mean of 2 pigs, with the absolute values indicated under “range”.

## Discussion

The current PRRSV vaccines on the market are of limited efficacy and come with several drawbacks [32]. Numerous efforts have been invested and strategies proposed for the development of novel efficacious PRRSV vaccines based on the antigenic properties of the PRRSV structural proteins and on the immunobiology of the virus [32, 34, 59]. Replicon vectors represent an elegant strategy to avoid the biological risks of virus spread and to circumvent potential immune modulating properties of modified live PRRSV vaccines [40]. Therefore, we explored the immunogenic and protective potential of VSV replicon particles as a vectored vaccine approach against PRRSV. Such VRP were successfully used before as efficacious experimental vaccines against SARS, IAV and BTV [48–52]. In the present study, VSV replicons were engineered to express PRRSV structural proteins, individually or in combination (Table 1). Emphasis was on GP5, M, GP4 and GP3, since these proteins are important targets for neutralizing and protective antibodies [11, 23, 26, 27], with the major neutralizing epitopes residing on GP5 [18]. Since several studies have shown that co-expression of PRRSV antigens resulted in better humoral and cellular immune responses than expression of the individual proteins [30, 60], GP5, M, GP4, GP3 and GP2 proteins were co-expressed to partly mimic the formation of the GP5/M and GP4/3/2 oligomers.

Vaccination of pigs with VRP expressing two or five PRRSV envelope proteins in total did not result in any significant reduction of viremia compared with the control group. A tendency for reduced PRRSV-related inflammatory responses was observed only when all five proteins were expressed. Interestingly, vaccination with GP5/M induced fever up to 41.5 °C for 1 day following challenge infection with PRRSV Olot/91 whereas mock-vaccinated pigs remained asymptomatic. Fever was less pronounced when all envelope proteins were included in the vaccine. Enhanced disease following vaccination with recombinant GP5 and M was observed previously [42, 61]. This was attributed to antibody-dependent enhancement of disease during natural infection, which is probably mediated by non-neutralizing antibodies [26, 62]. Such antibodies were shown to increase virus infection in porcine alveolar macrophage cultures and in vivo [62] involving different FcγR isoforms [63, 64].

Neutralizing antibodies were not found at any time following vaccination and challenge infection. Nevertheless, the vaccinated pigs seroconverted 15–17 days earlier than the mock-vaccinated animals after challenge virus infection, indicating that immunization with the recombinant VRP primed the immune system. In naïve pigs, the delayed seroconversion against the envelope proteins as opposed to the N protein after PRRSV infection or after immunization with vectored vaccines is well documented [31, 40, 42, 59]. Of note, with the MARC-145-adapted Olot/91 virus used in this study, seroconversion against N became detectable 9–14 days after challenge only (Tables 2, 3, 4) as opposed to the reported occurrence of anti-N antibodies as early as 5 to 7 days after PRRSV infection [31, 65]. This is probably related to the low level and short duration of replication of the MARC-145-adapted virus in pigs (Figures 5C, D, 6C, D). Despite the poor replication of this virus in vivo, vaccination of pigs with VRP did not result in any significant reduction of viremia. A better immunogenicity of PRRSV proteins was reported when the antigens were modified, coupled to immunostimulatory molecules or administered as purified proteins along with adjuvants [66, 67]. This raises the question whether the immunogenicity of the PRRSV envelope proteins expressed from VRP is affected by glycosylation, subcellular localization, intracellular retention or low stability. In order to elaborate on this, GP3 was modified with the aim of obtaining secreted GP3 by replacing the leader sequence with a canonical Igκ signal sequence followed by an optimal signalase cleavage site and by deleting the hydrophobic transmembrane anchor. However, the modified GP3 was retained in the cell, and no seroconversion against GP3ecto was obtained in absence of challenge virus infection. The lack of GP3 secretion may be related to the fact that GP3 is naturally retained in intracellular compartments despite the N-terminal signal sequence [11].

In order to address the question whether PRRSV structural proteins are immunogenic at all when expressed by VSV VRP in pigs, a vector expressing PRRSV N protein was included in one vaccination trial. N was chosen because it is the most immunogenic structural protein after PRRSV infection [31, 65]. Indeed, VSV*ΔG(N) was the only VRP capable of inducing a detectable antibody response in the absence of a viral challenge (Table 4). The co-vaccination experiments with VRP expressing IAV and PRRSV envelope proteins corroborated these results. The IAV proteins were strongly immunogenic while the PRRSV envelope proteins were not, clearly demonstrating that the VSV vector per se is functional in pigs. Although several vector vaccines failed to induce a protective immune response, immunizations essentially primed the immune system to a challenge infection [41, 42]. These reports and the present study altogether suggest that the PRRSV envelope proteins are expressed in a way to hide partially from the immune system. Whether this immune evasion strategy is due solely to glycan shielding [68] or to a particular intracellular topology remains to be investigated. Future efforts in vectored vaccine development should consider these aspects to enhance the immunogenicity of the PRRSV antigens.
